# A new association test based on disease allele selection for case–control genome-wide association studies

**DOI:** 10.1186/1471-2164-15-358

**Published:** 2014-05-12

**Authors:** Zhongxue Chen

**Affiliations:** Department of Epidemiology and Biostatistics, School of Public Health, Indiana University Bloomington, 1025 E. 7th street, PH C104, Bloomington, IN 47405 USA

**Keywords:** Generalized genetic model, Robust test, Single-nucleotide polymorphism

## Abstract

**Background:**

Current robust association tests for case–control genome-wide association study (GWAS) data are mainly based on the assumption of some specific genetic models. Due to the richness of the genetic models, this assumption may not be appropriate. Therefore, robust but powerful association approaches are desirable.

**Results:**

In this paper, we propose a new approach to testing for the association between the genotype and phenotype for case–control GWAS. This method assumes a generalized genetic model and is based on the selected disease allele to obtain a p-value from the more powerful one-sided test. Through a comprehensive simulation study we assess the performance of the new test by comparing it with existing methods. Some real data applications are also used to illustrate the use of the proposed test.

**Conclusions:**

Based on the simulation results and real data application, the proposed test is powerful and robust.

**Electronic supplementary material:**

The online version of this article (doi:10.1186/1471-2164-15-358) contains supplementary material, which is available to authorized users.

## Background

In a case–control genome-wide association study (GWAS), to detect the associated single-nucleotide polymorphisms (SNPs), we need to conduct a test for each individual SNP data, which are summarized as a 2-by-3 table. Although Pearson’s chi-square test can be used, it is usually less powerful than the Cochran-Armitage trend test (CATT) if the genetic model is known [1–3]. However, if the genetic models are unknown or various, the optimal scores in the CATTs are difficult or unable to find. If we use a CATT with fixed scores for all SNPs, we may lose power for some situations [4–13]. To circumvent this disadvantage, in the literature, many robust association tests have been proposed [7, 12, 14–22]. Those tests do not rely solely on one specific genetic model; rather they consider several possible genetic models simultaneously. In addition, many of them are based on the assumption that the underlying genetic model is one of the following three: additive, recessive, and dominant. For example, the maxmin efficiency robust test (MERT) by Gastwirth [23, 24], and the maximum of the three optimal CATTs under recessive, additive, and dominant models (MAX3) have been studied [6]. Zheng and Ng proposed a two-phase procedure called genetic model selection (GMS) method [12] which selects a genetic model from the three models in its first stage. On the contrary, Joo et al. proposed a test which eliminates genetic models [25]. Due to the environmental interaction, there are unlimited genetic models besides the three ideal models (recessive, additive, and dominant). Chen and Ng proposed a robust association test based on the generalized genetic model (GGM) [19], which includes the recessive, additive, and dominant models as special cases. Their approach obtains a p-value from a one-sided test for each of the two possible disease alleles. With the uncertainty of the disease allele, the overall p-value is then approximated from the two dependent tests.

In this paper, we propose a new robust association test which utilizes the GGM and obtains a one-sided p-value based on the selected disease allele. The performance of the new test is compared with existing methods in terms of controlling type I error rate and detecting power. Some real data applications are also used to demonstrate the use of the new test.

## Methods

### GGM and existing methods

The data of a diallelic SNP with alleles *A* and *a* in a case control GWAS are summarized in Table 1, where r_1_, r_2_, and r_3_ are the frequencies of genotypes *AA, Aa,* and *aa*, respectively, for the r cases; and s_1_, s_2_, and s_3_ are the frequencies of genotypes *AA*, *Aa*, and *aa*, respectively, for the s controls.

**Table 1:** **SNP data in a case control GWAS**

Genotype	***AA***	***Aa***	***aa***	Total
Case	*r*_*1*_	*r*_*2*_	*r*_*3*_	*r*
Control	*s*_*1*_	*s*_*2*_	*s*_*3*_	*s*
Total	*n*_*1*_	*n*_*2*_	*n*_*3*_	*n*

Among the three genotypes *AA, Aa*, and *aa*, the relative risks of genotypes *Aa* and *aa* to *AA* are defined as:1

Under the null hypothesis that there is no association between the genotype and phenotype, we have Λ_1_ = Λ_2_ = 1. Regarding the alternative hypothesis, we assume the underlying genetic model is a GGM, which is also called order-restricted relative risks model [19]. For the case where *a* is the disease allele, GGM assumes Λ_1_ ≥ 1 and Λ_2_ ≥ Λ_1_ with at least one of the inequalities is strictly greater than. It is easy to see that the aforementioned ideal models, recessive (Λ_1_ = 1, Λ_2_ > Λ_1_), additive (Λ_1_ = (1 + Λ_2_)/2), and dominant (Λ_1_ = Λ_2_ > 1), are all special cases of the generalized model. If *A,* rather *a,* is the disease allele, GGM assumes 1 ≥ Λ_1_ and Λ_1_ ≥ Λ_2_ with at least one of the inequalities is strict.

Suppose the frequencies for *AA, Aa*, and *aa* are *p*_*1*_*, p*_*2*_*, p*_*3*_ for cases and *q*_*1*_*, q*_*2*_, and *q*_*3*_ for controls, respectively. Under the null hypothesis that there is no association between the disease and the genotype, it is easy to show *p*_*1*_ *= q*_*1*_, *p*_*2*_ *= q*_*2*_, and *p*_*3*_ *= q*_*3*_. In this paper, we assume the cases and controls follow trinomial distributions TN(r, *p*_*1*_*, p*_*2*_*, p*_*3*_) and TN (s, *q*_*1*_*, q*_*2*_*, q*_*3*_), respectively.

The test statistics for some well-known existing methods are summarized as follows:

The CATT test statistic is [8]:

They are the scores assigned to the three columns.

The statistic for MAX3 is [6]:

The statistic for GMS is [12, 13]:

where *I* is the indicator function, and the Hardy-Weinberg disequilibrium trend test (HWDTT) statistic is given by [15]:

 and *c* is a constant and usually chosen as 1.645. Here n_i_ = s_i_ + r_i_ (i = 1,2,3), and n = n_1_ + n_2_ + n_3_.

The statistic for MERT is [24]:

### The proposed test

Let , it can be shown that under the null hypothesis of no association, the mean of the vector *E*[*T*] = 0, and variance-covariance matrix is.

We define the following “standardized” statistics:2

It is easy to prove the following result.

**Theorem 1.** Asymptotically, under the null hypothesis the statistics in (2) follow a multivariate normal distribution, *Z* ~ *MVN* (0, Σ_*Z*_), where the variance-covariance matrix is.

From theorem 1,  *Z*_1_, *Z*_2_ and *Z*_3_ are linear dependent; it is not difficult to show that asymptotically  *Z*_3_ = *aZ*_1_ + *bZ*_2_, where , and . It can also be shown that under the GGM, if *a* (or *A*) is the disease allele, the expectations of Z_i_ (i = 1,2,3) in (2) are all greater (or less) than 0. In addition, under the GGM, if z_1_ is close to 0, the genetic model is close to the recessive model. On the other hand, the genetic model is unlikely to be the recessive model if z_1_ is far from 0.

We will select the disease allele based on the sign of Z_3_, and combine the two one-sided p-values obtained based on the selected disease allele. Following the idea of combining p-values from independent studies using robust test [26], we define the test statistic as follows:3

where Φ(∙) is the cumulative density function (CDF) of the standard normal distribution (N(0,1)) and F^- 1^(∙) is the inverse of the CDF of the chi-square distribution with one degree of freedom.

Usually it is difficult to directly calculate the p-value (or critical value) for statistic C in (3). However, the p-value can be easily estimated using resampling method. Specifically, we first simulate two independent samples, z_1_, and z_2_, both from N(0,1). Then we calculate , where ,  are the estimates of *a* and *b*, and  are the estimates for *p*_*i*_ (*i* = 1, 2, 3) from the data. Next we calculate.

. We repeat the above steps K times and get K values for g. The p-value can then be estimated as (*number of g* ' *s that are greater than or equal to c*)/*K*, where c is the observed statistic calculated from data using (3).

## Results and discussion

### Simulation study

In this section, we will assess the performance of the proposed test by comparing it with existing methods in terms of controlling type I error rate and detecting power through a comprehensive simulation study. In the simulation study we assume that cases and controls in Table 1 follow trinomial distributions with probabilities *p = (p*_*1*_*,p*_*2*_*,p*_*3*_*)* and *q = (q*_*1*_*,q*_*2*_*,q*_*3*_*)*, respectively. It can be shown that, for given *q*_*i*_’s, and relative risks Λ_1_, Λ_2_, the values of the corresponding *p*_*i*_’s can be obtained as follows [17–21]: , , and .

We first assume Hardy–Weinberg equilibrium (HWE) holds for controls, and the minor allele frequencies (MAF) are 0.3 and 0.5. The disease allele is either the minor or the major allele. The numbers of cases (*r*) and controls (*s*) are both set to be 1000. Different pairs of *Λ*_1_ and *Λ*_2_ are used in the simulation to compare the performance of our proposed method with those of GMS, MERT, MAX3, Pearson’s chi-square test, and CATT with *x* = 0.5, which is the commonly used test under the assumption of additive genetic model. More specifically, we fix *Λ*_2_ to be 1.4 and let *Λ*_1_ vary from 1.0 to 1.4 with increment 0.1. Therefore, the three special genetic models are included in the simulation study. To assess the robustness of the proposed test, we also simulate data from genetic models other than the GGM: over-dominant model (*Λ*_1_ = 1.5, *Λ*_2_ = 1.4 ) and under-dominant model (*Λ*_1_ = 0.9, *Λ*_2_ = 1.4). The significance level is set to be 0.05, and the type I error rate and power are estimated by the proportions of rejections from 1000 replicates. To estimate the p-value for the proposed test, we resample 10,000 times (i.e., K = 10,000). The p-values from MAX3, GMS, and MERT are obtained by using the R package “Rassoc” [13].

When the null hypothesis is true (*Λ*_1_ = *Λ*_2_ = 1), all methods have rejection proportions close to the preset significance level 0.05 (For power values, see Additional file 1: Power plots of Figures S1-S5 for Tables 2-6); this indicates that they can control type I error rate. Table 2 reports the empirical powers of each method when HWE holds for controls with MAF equals to 0.3, and the minor allele is the risk allele. The chi-square test is usually less powerful than the proposed test except for the condition where the genetic model is over-dominant, under which the proposed test is more powerful than other methods. The CATT and MERT perform relatively poorly when the genetic models are far from the additive model. MAX3 and GMS perform similarly and have comparable power values with the proposed test; while the new test performs better when the genetic model is dominant or over-dominant. Table 3 lists the power values when HWE holds for controls with MAF equals to 0.3, and the major allele is the risk allele. Once again, the CATT and MERT lose power dramatically compared to other methods when the genetic model is recessive or under-dominant. The proposed test is more powerful than the MAX3 and GMS when the genetic models are close to recessive model. The chi-square test is less powerful than the proposed test when the genetic models are between recessive and dominant.

**Table 2:** **Empirical type I error rates and powers for each method from 1000 replicates at significance level 0.05 when the sample sizes are 1000 for cases and controls and HWE holds for controls with minor allele is the disease allele and MAF equals 0.3**

***Λ***_1_ ***Λ***_2_	(1,1)	(1, 1.4) RM*	(1.1, 1.4)	(1.2, 1.4) AM*	(1.3, 1.4)	(1.4, 1.4) DM*	(1.5,1.4) ODM*	(0.9, 1.4) UDM*
ChiSQ	0.053	0.927	0.759	0.528	0.395	0.428	0.524	0.992
MAX3	0.051	0.941	0.794	0.568	0.438	0.445	0.484	0.994
GMS	0.05	0.933	0.772	0.577	0.431	0.432	0.459	0.993
CATT	0.05	0.934	0.807	0.644	0.427	0.25	0.136	0.986
MERT	0.051	0.879	0.744	0.642	0.479	0.377	0.261	0.938
GGM	0.053	0.944	0.793	0.598	0.447	0.432	0.412	0.995
New	0.049	0.927	0.761	0.574	0.455	0.46	0.512	0.982

*RM: Recessive Model; *AM*: Additive Model; *DM*: Dominant Model; *ODM*: Over-dominant Model; *UDM*: Under-dominant Model.

**Table 3:** **Empirical type I error rates and powers for each method from 1000 replicates at significance level 0.05 when the sample sizes are 1000 for cases and controls and HWE holds for controls with major allele is the disease allele and MAF equals 0.3**

***Λ***_1_ ***Λ***_2_	(1,1)	(1, 1.4) RM*	(1.1, 1.4)	(1.2, 1.4) AM*	(1.3, 1.4)	(1.4, 1.4) DM*	(1.5,1.4) ODM*	(0.9, 1.4) UDM*
ChiSQ	0.046	0.538	0.496	0.615	0.805	0.933	0.988	0.752
MAX3	0.048	0.552	0.547	0.662	0.831	0.948	0.987	0.676
GMS	0.05	0.547	0.536	0.648	0.827	0.933	0.986	0.681
CATT	0.048	0.375	0.555	0.716	0.854	0.926	0.963	0.195
MERT	0.042	0.45	0.587	0.685	0.811	0.853	0.911	0.324
GGM	0.052	0.523	0.55	0.682	0.839	0.944	0.987	0.614
New	0.050	0.526	0.551	0.678	0.839	0.942	0.971	0.602

*RM: Recessive Model; *AM*: Additive Model; *DM*: Dominant Model; *ODM*: Over-dominant Model; *UDM*: Under-dominant Model.

Table 4 gives the empirical powers when HWE holds for controls with MAF equals to 0.5. As seen in Tables 2 and 3, the CATT and MERT are less powerful when the genetic model is dominant or over-dominant. On the contrary, chi-square test is reasonably powerful under those situations. The proposed test has the largest powers when the genetic model is close to the dominant one (i.e.,  *Λ*_1_ = 1.3, 1.4, 1.5). For other situations, the new test has comparable powers with those from MAX3 and GMS.

**Table 4:** **Empirical type I error rates and powers for each method from 1000 replicates at significance level 0.05 when the sample sizes are 1000 for cases and controls and HWE holds for controls MAF equals 0.5**

***Λ***_1_ ***Λ***_2_	(1,1)	(1, 1.4) RM*	(1.1, 1.4)	(1.2, 1.4) AM*	(1.3, 1.4)	(1.4, 1.4) DM*	(1.5,1.4) ODM*	(0.9, 1.4) UDM*
ChiSQ	0.048	0.854	0.736	0.646	0.697	0.81	0.909	0.973
MAX3	0.045	0.868	0.778	0.704	0.732	0.818	0.909	0.962
GMS	0.044	0.857	0.768	0.692	0.724	0.81	0.908	0.966
CATT	0.044	0.795	0.787	0.761	0.724	0.705	0.701	0.815
MERT	0.044	0.789	0.782	0.763	0.729	0.718	0.722	0.806
GGM	0.045	0.865	0.795	0.735	0.746	0.816	0.901	0.962
New	0.041	0.84	0.773	0.714	0.747	0.829	0.919	0.952

*RM: Recessive Model; *AM*: Additive Model; *DM*: Dominant Model; *ODM*: Over-dominant Model; *UDM*: Under-dominant Model.

Tables 5 and 6 report the empirical powers when HWE does not hold with the genotypic frequencies for controls are (0.1, 0.36, 0.54), and the disease risks are minor allele and major allele, respectively. Once again we can see that CATT and MERT are not robust; they may lose power dramatically under some conditions (e.g., over-dominant in Table 5 and under-dominant in Table 6). The proposed test has comparable power values with those from the MAX3 and GMS; under some situations, it is more powerful (e.g., over-dominant model in Table 5).

**Table 5:** **Empirical type I error rates and powers for each method from 1000 replicates at significance level 0.05 when the sample sizes are 1000 for cases and controls and the genotype frequencies for controls are (0.1,0.36,0.54) with minor allele is the disease allele**

***Λ***_1_ ***Λ***_2_	(1,1)	(1, 1.4) RM*	(1.1, 1.4)	(1.2, 1.4) AM*	(1.3, 1.4)	(1.4, 1.4) DM*	(1.5,1.4) ODM*	(0.9, 1.4) UDM*
ChiSQ	0.046	0.93	0.743	0.539	0.426	0.481	0.565	0.995
MAX3	0.04	0.944	0.771	0.601	0.473	0.486	0.507	0.995
GMS	0.04	0.929	0.756	0.609	0.471	0.49	0.517	0.992
CATT	0.043	0.936	0.807	0.659	0.459	0.314	0.194	0.974
MERT	0.044	0.88	0.763	0.656	0.517	0.409	0.302	0.933
GGM	0.043	0.952	0.786	0.635	0.484	0.46	0.437	0.995
New	0.049	0.926	0.755	0.616	0.485	0.514	0.556	0.988

*RM: Recessive Model; *AM*: Additive Model; *DM*: Dominant Model; *ODM*: Over-dominant Model; *UDM*: Under-dominant Model.

**Table 6:** **Empirical type I error rates and powers for each method from 1000 replicates at significance level 0.05 when the sample sizes are 1000 for cases and controls and the genotype frequencies for controls are (0.1,0.36,0.54) with major allele is the disease allele**

***Λ***_1_ ***Λ***_2_	(1,1)	(1, 1.4) RM*	(1.1, 1.4)	(1.2, 1.4) AM*	(1.3, 1.4)	(1.4, 1.4) DM*	(1.5,1.4) ODM*	(0.9, 1.4) UDM*
ChiSQ	0.042	0.55	0.553	0.673	0.812	0.927	0.981	0.749
MAX3	0.044	0.568	0.6	0.718	0.843	0.939	0.987	0.712
GMS	0.041	0.585	0.61	0.718	0.82	0.922	0.98	0.727
CATT	0.041	0.431	0.607	0.765	0.854	0.918	0.962	0.248
MERT	0.042	0.512	0.635	0.755	0.808	0.866	0.896	0.385
GGM	0.036	0.55	0.611	0.743	0.848	0.942	0.986	0.66
New	0.043	0.552	0.597	0.734	0.849	0.941	0.981	0.654

*RM: Recessive Model; *AM*: Additive Model; *DM*: Dominant Model; *ODM*: Over-dominant Model; *UDM*: Under-dominant Model.

The method based on GGM by Chen and Ng (GGM in Tables 2–6) has similar performance as the proposed test. However, if the disease allele is the minor allele, the new test is more powerful than the GGM method when the genetic model is dominant or over-dominant (see Tables 2, 4 and 5). In addition, unlike the proposed test, the GGM method doesn’t report the disease allele.

### Real data application

To illustrate the use of the proposed test, we apply it to some real data. The SNP rs181489 has been shown to be associated with Parkinson disease [27]. Table 7 lists the genotypic counts of cases and controls of this SNP from fifteen sites [27]. We apply the proposed test and others to the data of each site to obtain p-values. Table 8 reports the results along with the three statistics, z_1_, z_2_ and z_3_. Out of the 15 populations, 14 have positive values for z_3_, indicating the disease allele is A rather than G for this SNP. Recall the statistic z_1_ compares genotype GG to GA between controls and cases. A small z_1_ indicates the relative risk *Λ*_1_ is close to 1, and therefore the genetic model is close to the recessive one. On the other hand, if z_1_ is large, the underlying genetic model is unlikely to be a recessive model. For population D, the estimated z_1_ is -0.090, the genetic model is close to a recessive one, under which the CATT test is less powerful than other robust methods. In fact, for this population CATT obtains the largest p-value (0.024). Similar observation can be found for population G. In contrast, z_1_ and z_2_ from populations H and O are both large, indicating the underlying genetic models are close to the additive model, under which the CATT is more powerful than others. Therefore, there is no surprise that CATT obtains the smallest p-values from those two populations.

**Table 7:** **Genotypic count data for rs181489 from different populations (data obtained from**[27]**)**

Population	Case			Control			Total n
	GG	GA	AA	GG	GA	AA	
A: Australia	400	402	99	320	307	58	1586
B: France	244	245	57	5	61	11	623
C: Germany	86	119	19	16	18	7	265
D: Germany	222	176	85	133	107	25	748
E: Germany	144	149	39	169	140	29	670
F: Greece	44	67	17	44	37	10	219
G: Greece	119	126	47	130	123	18	563
H: Ireland	140	147	58	229	157	38	769
I: Italy	78	86	21	87	71	9	352
J: Italy	73	88	28	44	43	8	284
K: Italy	33	47	10	41	21	9	161
L: Norway	290	233	80	240	228	56	1127
M: Poland	158	144	47	171	135	30	685
N: Sweden	50	30	10	91	68	17	266
O: USA	156	170	50	191	137	32	736

**Table 8:** **P-values and Z statistics from different methods based on each population of the SNP rs181489 data**

Population	ChiSQ	MAX3	GMS	CATT	MERT	New	Z1	Z2	Z3
A: Australia	0.23	0.19	0.20	0.14	0.11	0.16	0.44	1.66	1.72
B: France	0.66	0.64	0.47	0.41	0.38	0.51	0.34	0.84	0.90
C: Germany	0.20	0.18	0.51	0.46	0.31	0.23	0.54	-1.70	-1.41
D: Germany	0.011	0.0060	0.0055	0.024	0.014	0.0088	-0.090	3.02	2.82
E: Germany	0.16	0.11	0.089	0.055	0.058	0.099	1.36	1.36	1.69
F: Greece	0.11	0.080	0.12	0.076	0.11	0.082	2.02	0.51	1.19
G: Greece	0.0018	0.0011	0.0011	0.0032	0.0013	0.0012	0.63	3.51	3.52
H: Ireland	1.2e-4	5.8e-5	5.6e-5	2.2e-5	2.3e-5	7.0e-5	2.70	3.28	3.94
I: Italy	0.055	0.043	0.036	0.020	0.016	0.033	1.35	2.00	2.29
J: Italy	0.23	0.19	0.15	0.098	0.088	0.15	0.79	1.53	1.71
K: Italy	0.013	0.018	0.015	0.070	0.15	0.012	2.94	-0.31	0.63
L: Norway	0.18	0.34	0.25	0.94	0.73	0.43	-1.31	1.33	0.86
M: Poland	0.12	0.10	0.081	0.049	0.041	0.077	0.88	1.88	2.05
N: Sweden	0.69	0.79	0.80	0.78	0.89	0.96	-0.78	0.37	0.16
O: USA	0.0048	0.0031	0.0029	0.0013	0.0020	0.0026	2.66	1.90	2.61

## Conclusions

Although CATT has been widely used in case–control GWAS with the assumption that the underlying genetic model is additive, its performance may be very poor if the true genetic model is not additive. Therefore, robust but powerful association tests are more appropriate when detecting the associated SNPs. Many existing association tests make the assumption that the genetic model is one of the three special genetic models (recessive, additive, and dominant), which may be a too strong assumption in practice. In this paper, we propose a robust association test without making strong assumption about the genetic model. Our simulation results show that even the assumption of GGM is violated (e.g., over- and under- dominant models), the proposed test still has reasonable power; indicating it is a robust test. In terms of computational cost, the proposed test is reasonably fast. For instance, it took my desktop about 70 seconds to get the results in Table 8 for the real data application. Our simulation study also confirmed that the proposed test can control type I error rate with smaller cutoff p-value, e.g., 10^-4^ and 10^-5^ (see Additional file 2: Table S1-S2).

The test statistic in (3) is defined based on the idea of combining p-values from independent studies using chi-square distribution with 1 degree of freedom [26]. Although there are many other approaches available in the literature [26, 28–31], it remains a research topic to choose the best one if there is any. However, it should be noticed that for case control GWAS, a robust method, such as the proposed test, is desirable due to the various underlying genetic models. In addition, when we combine the p-values from the 15 independent studies using the chi-square distribution with 1 degree of freedom [26], the overall p-value is 2.6 × 10^-11^.

Through simulation studies and real data applications, we have shown that the proposed test is robust and powerful. In addition, the three statistics, z_1_, z_2_, and z_3_, may also provide useful information about the disease allele and the genetic model.

## Electronic supplementary material

Additional file 1: **Power plots of Figures S1-S5 for Tables 2-6.** (PDF 76 KB)

Additional file 2: Table S1: Empirical type I error rate (×10^-4^) for each method from 10^6^ replicates at significance level 10^-4^ with the sample sizes 1000 for cases and controls and given genotype frequencies for controls. **Table S2.** Empirical type I error rate (×10^-5^) for each method from 10^6^ replicates at significance level 10^-5^ with the sample sizes 1000 for cases and controls and given genotype frequencies for controls. (DOCX 12 KB)

## Abbreviations

GWAS: Genome-wide association study
SNP: Single-nucleotide polymorphism
CATT: Cochran-Armitage trend test
MERT: Maxmin efficiency robust test
MAX3: The maximum of the three optimal
CATTs: Under recessive, additive, and dominant models
GMS: Genetic model selection
GGM: Generalized genetic model
CDF: Cumulative density function
HWDTT: Hardy-Weinberg disequilibrium trend test
HWE: Hardy–Weinberg equilibrium.
