# Supplementary figures and images for: A new association test based on disease allele selection for case–control genome-wide association studies
Source: BMC Genomics. 2014 May 12;15(1):358. doi: 10.1186/1471-2164-15-358 (PMC4059871; doi:10.1186/1471-2164-15-358)

Figure S2

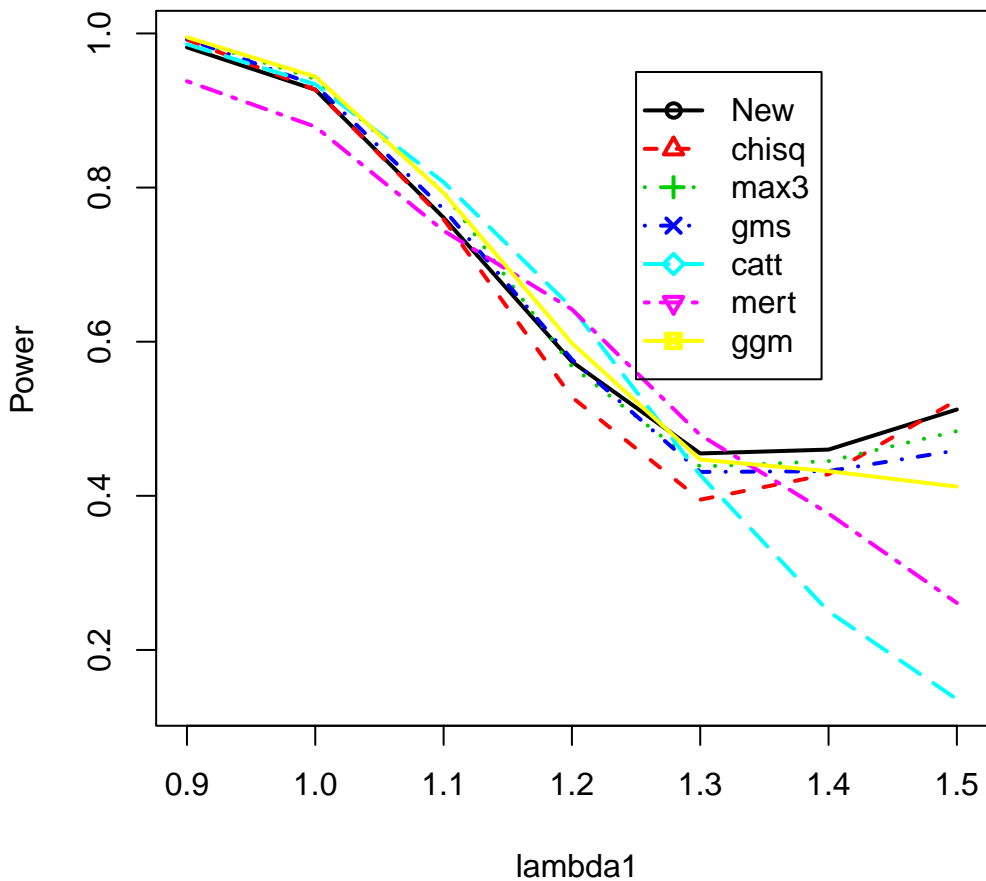

**Figure S3**

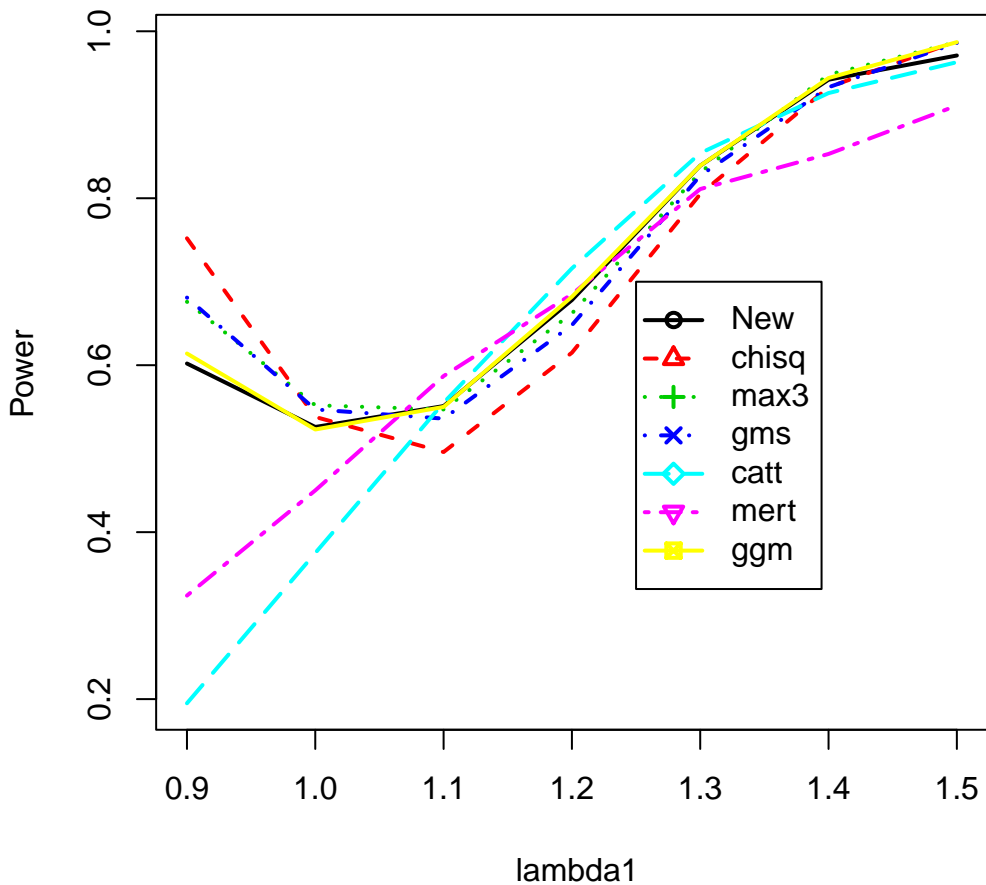

**Figure S4**

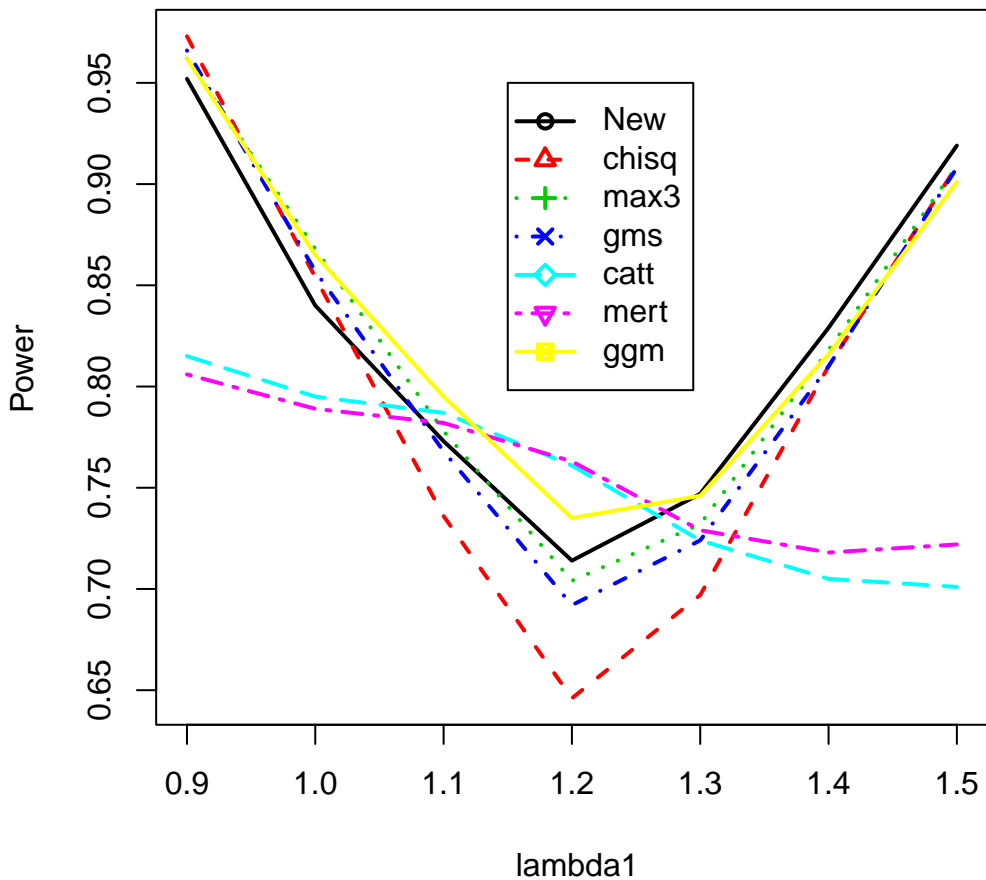

**Figure S5**

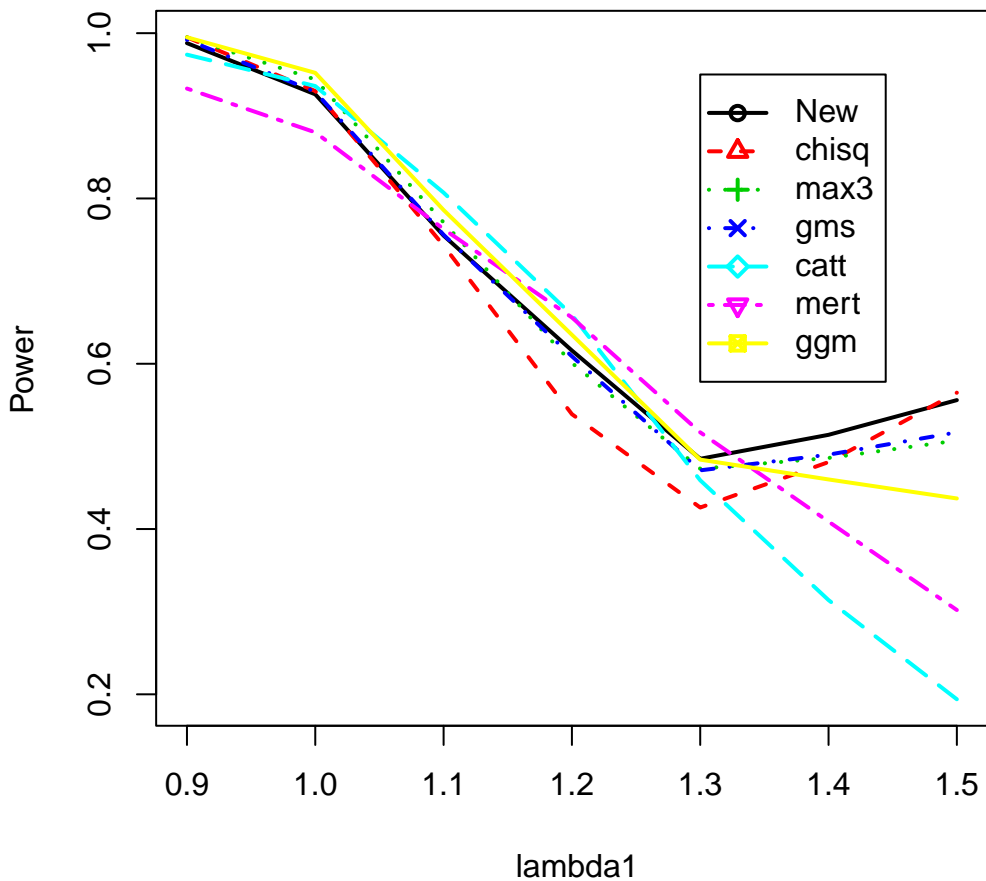

Figure S6

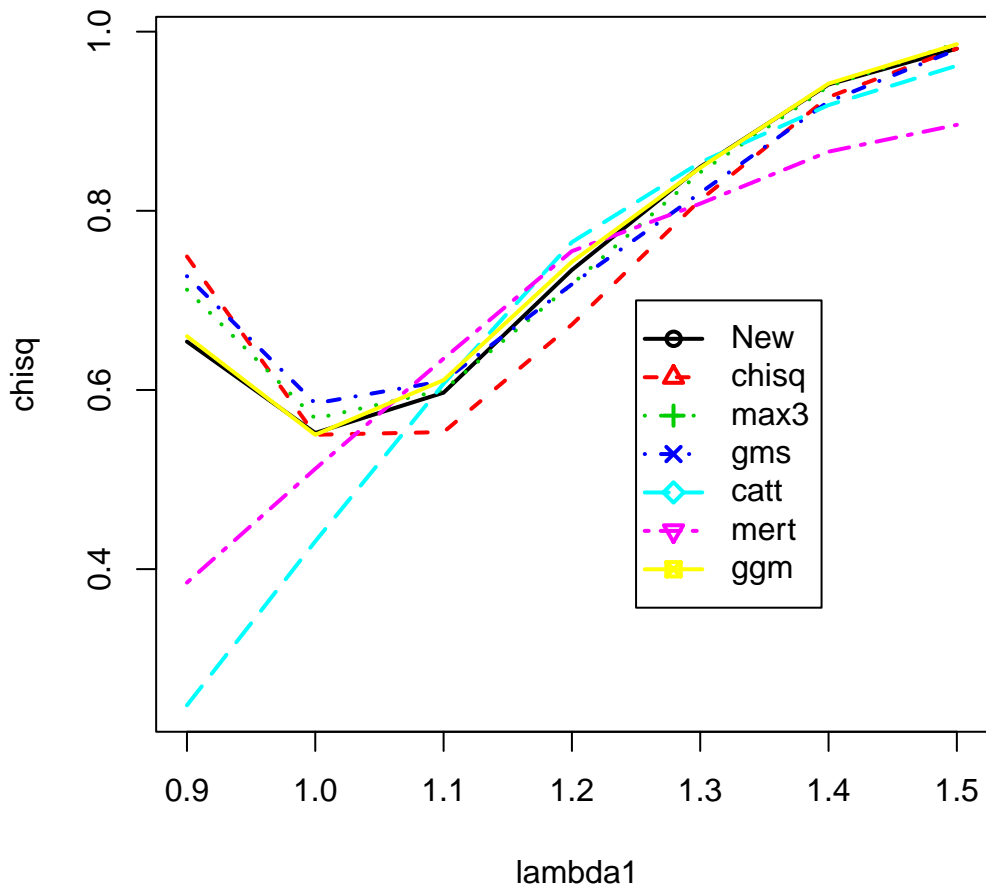

Supplement: Supplementary file 1 — Additional file 1: Power plots of Figures S1-S5 for Tables 2-6. (PDF 76 KB) [file 12864_2014_6111_MOESM1_ESM.pdf]
