# Supplementary material for: A new association test based on disease allele selection for case–control genome-wide association studies
Source: BMC Genomics. 2014 May 12;15(1):358. doi: 10.1186/1471-2164-15-358 (PMC4059871; doi:10.1186/1471-2164-15-358)
Supplement: Supplementary file 2 — Additional file 2: Table S1: Empirical type I error rate (×10-4) for each method from 106 replicates at significance level 10-4 with the sample sizes 1000 for cases and controls and given genotype frequencies for controls. Table S2. Empirical type I error rate (×10-5) for each method from 106 replicates at significance level 10-5 with the sample sizes 1000 for cases and controls and given genotype frequencies for controls. (DOCX 12 KB) [file 12864_2014_6111_MOESM2_ESM.docx]

Supplementary Table 1. Empirical type I error rate (×10^-4^) for each method from 10^6^ replicates at significance level 10^-4^ with the sample sizes 1000 for cases and controls and given genotype frequencies for controls.

| genotype frequencies | 0.01, 0.18,0.81 | 0.09,0.42,0.49 | 0.25,0.50,0.25 | 0.1,0.36,0.54 |
| --- | --- | --- | --- | --- |
| ChiSQ | 0.72 | 1.13 | 1.05 | 1.00 |
| MAX3 | 0.78 | 1.27 | 1.07 | 0.96 |
| GMS | 0.80 | 1.13 | 1.04 | 0.91 |
| CATT | 1.00 | 1.07 | 0.87 | 1.07 |
| MERT | 0.92 | 1.05 | 0.89 | 1.12 |
| GGM | 0.84 | 1.16 | 0.93 | 1.02 |
| New | 0.74 | 1.21 | 0.95 | 1.01 |

Supplementary Table 2. Empirical type I error rate (×10^-5^) for each method from 10^6^ replicates at significance level 10^-5^ with the sample sizes 1000 for cases and controls and given genotype frequencies for controls.

| genotype frequencies | 0.01, 0.18,0.81 | 0.09,0.42,0.49 | 0.25,0.50,0.25 | 0.1,0.36,0.54 |
| --- | --- | --- | --- | --- |
| ChiSQ | 0.7 | 0.8 | 0.9 | 1.6 |
| MAX3 | 0.8 | 1.3 | 0.9 | 1.7 |
| GMS | 0.6 | 1.2 | 1.0 | 1.4 |
| CATT | 1.5 | 1.2 | 1.2 | 0.9 |
| MERT | 0.8 | 0.8 | 1.2 | 1.2 |
| GGM | 0.5 | 1.2 | 1.2 | 1.6 |
| New | 0.6 | 1.3 | 0.9 | 1.4 |
